# Synthesis and Antiplatelet Aggregation Activity Evaluation of some 2-Aminopyrimidine and 2-Substituted-4,6-diaminopyrimidine Derivatives

**Published:** 2015

**Authors:** Marjan Esfahanizadeh, Shohreh Mohebbi, Behnam Dasht Bozorg, Salimeh Amidi, Ali Gudarzi, Seyed Abdolmajid Ayatollahi, Farzad Kobarfard

**Affiliations:** a *Department of Medicinal Chemistry, School of Pharmacy,ShahidBeheshti University of Medical Sciences, Tehran, Iran.*; b *Department of Medicinal Chemistry, School of Pharmacy, Zanjan University of Medical Sciences, Zanjan, Iran.*; c *PhytochemistryResearch Center, ShahidBeheshti University of Medical Sciences, Tehran, Iran.*; d *Central Research Laboratories, ShahidBeheshti University of Medical Sciences, Tehran, Iran.*

**Keywords:** 2-aminopyrimidines, 2-Substituted-4, 6-diaminopyrimidines, Antiplatelet aggregation

## Abstract

A series of novel 2-aminopyrimidine and 2-Substituted-4,6-diaminopyrimidine derivatives have been synthesized and their antiplatelet aggregation activities were assessed against ADP and arachidonic acid-induced platelet aggregation in human plasma using light transmission aggregometry. Among the tested derivatives, compounds Ia, I_b_, I_B_ and II_16_ exhibited the highest antiplatelet aggregation activity (36.75, 72.4, 62.5 and 80 µM). None of the compounds showed satisfactory activity against the aggregation induced by ADP but acceptable activities were observed against the aggregation induced by arachidonic acid. 2- aminopyrimidines were more active than 4,6- diaminopyrimidines in this respect.

## Introduction

Platelets play an important role in maintaining cardiovascular integrity and in regulating the bleeding process by blood-clot formation (1). However, uncontrolled platelet aggregation is dangerous in arterial blockage and may lead to life threatening disorders (2). Antiplatelet agents are therefore considered as a significant tool in the treatment and/or prevention of cardiovascular thrombotic disease (3-5). Antiplatelet agents such as aspirin (acetylsalicylic acid), clopidogrel or ticlopidine and anticoagulants such as warfarin are currently two predominant groups of orally consumable drugs in standard therapeutic protocols for prophylaxis and treatment of venous thrombosis and reducing the risk of recurrent myocardial infarction (6-8).

Currently aspirin, which irreversibly inhibits cyclooxygenase I-mediated transformation of arachidonic acid to thromboxane A_2_ (TXA_2_), and the P_2_Y_12_ antagonists clopidogrel and prasugrel, which selectively and irreversibly bind to theP_2_Y_12 _ADP receptor are routinely used as antiplatelet agents (9, 10).

However there are still some serious limitations to these agents which include weak inhibition of platelet function (*eg*. aspirin) (11), slow onset of action (*eg*. clopidogrel) (12), variable response to treatment among the patients (*eg*. clopidogrel and aspirin) (11, 12) and high incidence of bleeding events which occur in both aspirin and clopidogrel drug therapy (13, 14). Considering the current situation, development of novel antiplatelet agents which are safe and effective is an urgent need (15).

Aminopyrimidine derivatives are an interesting group of compounds with various reported biological properties. Pyrimidine ring can be found in the structures of many important drugs such as nucleoside antibiotics, antibacterials and cardiovascular agents (16, 17).

**Figure 1:**
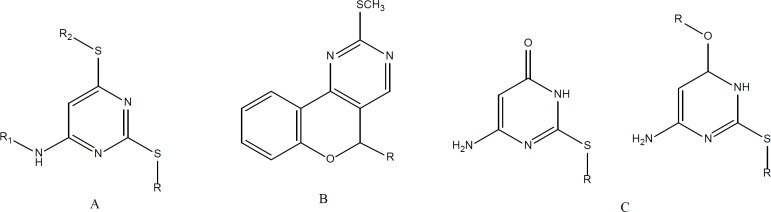
Active antiplatelet pyrimidine derivatives. A. 6-alkylamino-2,4-dialkyl(aryl)thiopyrimidines [7], R = Me,

Based on the hypothesis suggested by Cattaneo *et al*. (18), amino pyrimidine ring could be considered as a simplified form of the active metabolites of the thienopyridines and ATP derivatives. The active metabolites of thienopyridines have a simple monocyclic structure which implies that the presence of a bicyclic structure like that of a purine ring is not an absolute requirement for the affinity for the ADP receptor or so on platelet membrane.

**Figure 2:**
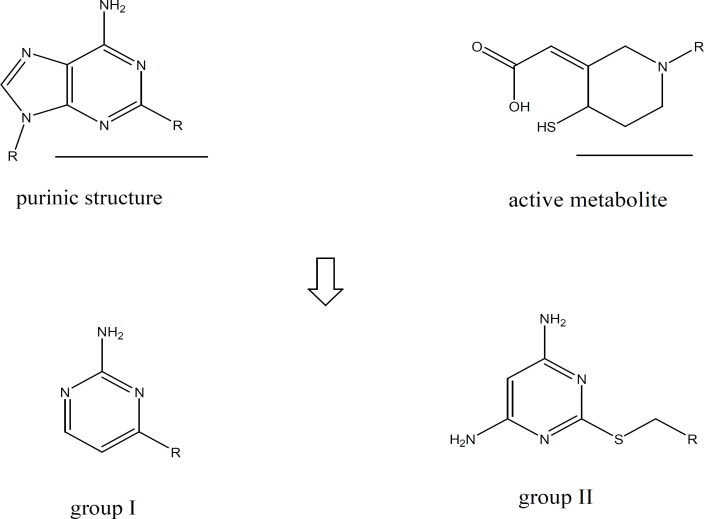
Comparing the structures of aminopyrimidines with the structures of purine and the active metabolite of clopidogrel.

A group of amino pyrimidine derivative with thioether substituents has been synthesized and evaluated by Cattaneo *et al*. The compound showed satisfactory anti platelet aggregation effects when ADP had been used for aggregation induction (18). Based on the mentioned reports and in order to investigate the capability of amino pyrimidine derivatives in inhibition of platelet aggregation pathways, we synthesized two groups of amino pyrimidines with the following structures: 

**Figure 3:**
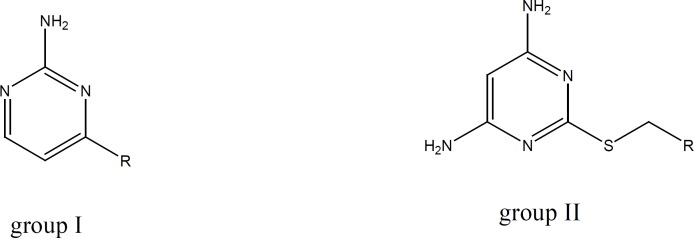
Chemical structure of the synthesized compounds

Comparing the activity of these compounds in inhibition of platelet aggregation induced by ADP and arachidonic acid will provide some insights into the structure activity relationship of these compounds.

*Chemistry*

**Figure 4:**
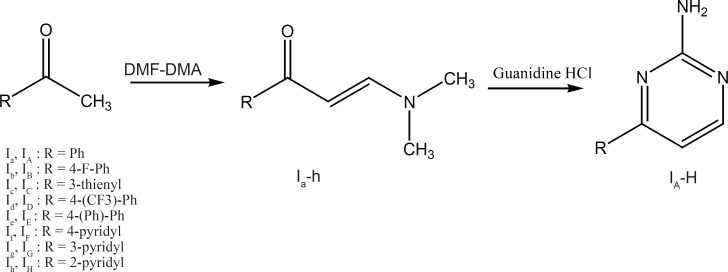
Compounds (I_a-h_) and (I_A-H_) synthesis scheme. Reagent and conditions: (a) DMF, reflux, 24 h; (b) NaOCH_3_, Isopropanol, reflux, 48 h.

**Figure 5:**
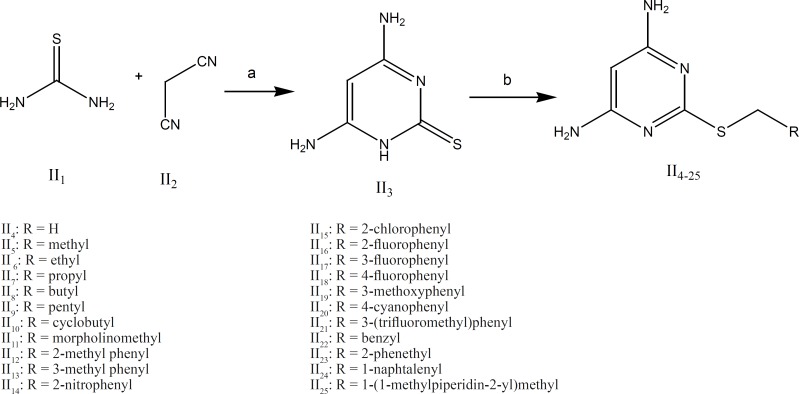
Compounds (II_4-25_) synthesis scheme. Reagents and conditions: (a) EtONa, reflux, 3 h; (b) NaOH 0.1 M, CH_3_OH, R-CH_2_-X (X = Cl, Br), rt, 18 h10).

The synthetic procedures for groups I and II are illustrated in Figures 4 and 5.

Group I: Methyl ketones (1a-h) were allowed to react with dimethylformamide-dimethylacetate (DMF-DMA) to produce 3-(dimethylamino)-1-aryl-2en-1-ones (2a-h). These intermediates could be then condensed with guanidine HCl to obtain the corresponding amino pyrimidine ring systems (19).

Group II: As it is shown in Figure 5 the intermediate II_3_ (4,6-diaminopyrimidine-2-thiol) was obtained by the reaction of thiourea and malononitrile in absolute ethanol as the solvent. Subsequent reaction of compound II_3_with various alkylhalides at room temperature afforded compounds II_4-25_ in good yields.

Structure confirmation of the synthesized intermediates and the final products was performed using IR, NMR and Mass spectrometry (20).

## Experimental

*General*

All evaporations were carried out in vacuo with a rotary evaporator. Melting points (˚C) were determined by capillary method on an electrothermal melting point apparatus. Infrared spectra were recorded as thin films of KBr plates with U_max_ in inverse centimeters. Nuclear magnetic resonance spectra for proton (^1^H NMR) were recorded on a Bruker DRX-Avance (500 MHz) spectrometer. Chemical shift values are expressed in ppm (parts per million) relative to tetramethylsilane (TMS) as internal standard; s: singlet, d: doublet, dd: double doublet, t: triplet, q: quartet, m: multiplet, br s: broad singlet. Thin layer chromatography (TLC) was performed on Whatman SilG/UV_254_ silica gel plates with fluorescent indicator and the spots were visualized under 254 and 366 nm illumination. Mass analyses were performed on an Agilent 6400 series equipped with an electrospray (ESI) ionization interface (drying gas adjusted at 300 ˚C, nebulizing gas flow at 12 L/min). All the compounds were analyzed for C, H, N and S on a Costech model 4010 and agreed with the proposed structures within ±0.4% of the theoretical values.

*General procedure for the preparation of compounds (I*_a-h_ *)*

The detailed description of the methods used for preparation of compounds I_a_to I_h _and compounds I_A _to I_H_ is reported in reference 19. Briefly to a solution of acetophenone (84 mmol) in DMF (16 mL), was added DMF-DMA (84 mmol) and the solution was heated under reflux for 24 h. Brine (25 mL) was added to the reaction mixture after cooling and the reaction mixture was then extracted with CH_2_Cl_2 _(3 × 25 mL). The combined organic fractions were dried over anhydrous Na_2_SO_4_ and the solvent was evaporated under reduced pressure. The residue was dissolved in EtOAc (12 mL), followed by addition of n-hexane (100 mL). The precipitate thus obtained was filtered and dried to give the pure product I_a _as a yellow solid (19).

*General procedure for the preparation of compounds (I*_A-H_ *)*

To a solution of compounds I_a_ (2.68 mmol) in isopropanol (13.5 mL) were added sodium methoxide (10.7 mmol) and guanidine hydrochloride (4.02 mmol) and the solution was heated under reflux for 48h. Distilled water (25 mL) was added to the reaction mixture after cooling and the mixture was then extracted with EtOAc (3 × 15 mL). The combined organic fractions were dried over anhydrous Na_2_SO_4_ and the solvent was evaporated under reduced pressure. The residue was dissolved in EtOAc (2 mL), followed by addition of *n*-hexane (25 mL). The precipitate thus obtained was filtered and dried to give I_A-H_.

*Representative procedure for the preparation of compounds (II*_4-25_ *)*

The detailed description of the methods used for preparation of compounds II_4_ to II_25_is reported in reference 20. Briefly, to a solution of 4,6-diaminopyrimidine-2-thiol (II_3_, 1.4 mmol) in methanol, was added alkyl halide (3.5 mmol) under basic conditions (NaOH 0.1 M, 14 mL). The mixture was then stirred for 18h at room temperature. After removing the solvent under reduced pressure, the residue was washed by water and the precipitate was collected as a solid. All compounds were obtained in acceptable purity and no further purification was needed (20).

## Results and Discussion

All the synthesized compounds were screened for their effects on human platelet aggregation induced by arachidonic acid and ADP using light transmission aggregometry. IC_50_ was determined as the concentration of the test compounds that exhibit platelet aggregation by 50%. The *in-vitro* antiplatelet activity of the synthesized derivatives is listed in Tables 1 and 2.

**Table 1:** Antiplatelet activity of group I derivatives

**Compound**	**R**	**A.A IC**_50_ **µM**	**ADP** **%** **inhibition**
Ia (1.25 mM)	ph		
I_A_ (0.75 mM)	ph		
Ib (1 mM)	4-F- ph		
I_B_ (2.5 mM)	4-F- ph		
I_c_ (1 mM)	3-thienyl		
I_C_ (1.6 mM)	3-thienyl		
I_d_ (1.3 mM)	4-(CF_3_)- ph		
I_D_ (0.5 mM)	4-(CF_3_)- ph		
Ie (1.3 mM)	4-( ph)- ph		
I_E_ (1 mM)	4-( ph)- ph		
If (1 mM)	4-pyridyl		
I_F _(1 mM)	4-pyridyl		
Ig (2.5 mM)	3-pyridyl		
I_G_ (0.5 mM)	3-pyridyl		
I_h_ (1.25 mM)	2-pyridyl		
I_H_ (1.25 mM)	2-pyridyl		
Indomethacin			
Aspirin			

Comparing the activities of the two aminopyrimidine groups indicates that none of the compounds showed satisfactory activity against the aggregation induced by ADP. Therefore it could be concluded that the compounds do not interfere with ADP receptors on platelet membrane. Howeveracceptable activities were observed in both groups against the aggregation induced by arachidonic acid. This is not in agreement with the report by Cattaneo *et al. *who introduced a group of aminopyrimidines as active platelet aggregation inhibitors which interfere with ADP receptors

**Table 2:** Antiplatelet activity of group II derivatives

**Compound** **(In 1 mM)**	**R**	**A.A IC**_50_ **µM**	**ADP % ** **inhibition**
II_4_	H		
II_5_	Methyl		
II_6_	Ethyl		
II_7_	Propyl		
II_8_	Butyl		
II_9_	Pentyl		
II_10_	Cyclobutyl		
II_11_	Morpholinomethyl		
II_12_	2-methylphenyl		
II_13_	3-methylphenyl		
II_14_	2-nitrophenyl		
II_15_	2-chlorophenyl		
II_16_	2-fluorophenyl	**80**	
II_17_	3-fluorophenyl		
II_18_	4-fluorophenyl		
II_19_	3-methoxyphenyl		
II_20_	4-cyanophenyl		
II_21_	3-(trifluoromethyl)phenyl		
II_22_	Benzyl		
II_23_	2-phenethyl		
II_24_	1-naphthalenyl		
II_25_	1-(1-methylpiperidin-2-yl)methyl		
Indomethacin			
aspirin			

Comparing the overall results obtained for aminopyrimidines I and II indicates that 2-aminopyrimidines (I) were more active than 4,6-diaminopyrimidines (II). Only compound 16 in group II showed satisfactory IC_50_ (80 µM).

Among the 2-aminopyrimidines group (I), on the other hand, a few compounds (I_a_, I_b_, I_B_ and I_G_) showed good activities (36.75, 72.4, 62.5 and 192 µM)Interestingly, compounds with fluorine substituent on phenyl ring (I_b_, I_B_) were among the most active compounds.

Global physicochemical properties for compounds I_a-h_, I_A-H _and II_4-25 _were calculated using Chemdraw Ultra, Chem3D Ultra version 8.0 and Hyper Chem professional and the results are presented in Tables 3 and 4.

Efforts to find a relationship between these physicochemical parameters and anti platelet aggregation activity of the compounds did not result in a clear correlation. This could be due to the fact that the compounds are different in their access to their targets in the platelet aggregation pathway induced by arachidonic acid. 

Further mechanistic studies are needed to clarify the mechanism of antiplatelet activity of these compounds.

**Table 3:** Global physicochemical properties for compounds group I.

**Compound**	**ClogP** a	**P ** b	**V** c	**SA** d	**D** e
Ia	2.2974				
I_A_	1.774				
Ib	2.516				
I_B_	1.926				
I_c_					
I_C_	1.4697				
I_d_	3.3127				
I_D_	2.6730				
Ie	4.185				
I_E_	3.66				
If	1.1964				
I_F _	0.409				
Ig	1.1964				
I_G_	0.1997				
I_h_	1.5964				
I_H_	0.409				

A ClogP were calculated by using Chem Draw Ultra version 8.0.

B Polarizability values were calculated by using Hyper Chem Professional.

C Molecular volume values were calculated by using Hyper Chem Professional.

D Surface area values were calculated by using Hyper Chem Professional.

e Dipole (debye) values were calculated by using Chem3D Ultra version 8.0.

**Table 4:** Global physicochemical properties for compounds group II.

**Compound**	**ClogP** A	**P** B	**V** C	**SA** D	**D** E
II_4_					
II_5_					
II_6_					
II_7_					
II_8_					
II_9_					
II_10_					
II_11_					
II_12_					
II_13_					
II_14_					
II_15_					
II_16_					
II_17_					
II_18_					
II_19_					
II_20_	1.867				
II_21_	3.317				
II_22_	2.963				
II_23_	3.342				
II_24_	3.608				
II_25_	2.397				

A ClogP were calculated by using Chem Draw Ultra version 8.0.

B Polarizability values were calculated by using Hyper Chem Professional.

C Molecular volume values were calculated by using Hyper Chem Professional.

D Surface area values were calculated by using Hyper Chem Professional.

e Dipole (debye) values were calculated by using Chem3D Ultra version 8.0.
